# Biofouling mechanism and cleaning procedures for *Spirulina platensis* as an organic fertilizer draw solution

**DOI:** 10.1007/s11356-023-28694-4

**Published:** 2023-07-21

**Authors:** Ghada Al Bazedi, Noha Soliman, Hani Sewilam

**Affiliations:** 1 Center for Applied Research on the Environment and Sustainability (CARES), School of Science and Engineering, The American University in Cairo, AUC Avenue, P.O. Box: 74, New Cairo, 11835 Egypt; 2 Chemical Engineering Department, Engineering and Renewable Energy Research Institute, 33 El-Bohouth St., Dokki, PO Box 12622, Giza, Egypt; 3 Department of Engineering Hydrology, RWTH Aachen University, Mies-van-der-Rohe Strasse 17, 52074 Aachen, Germany

**Keywords:** Forward osmosis, Organic fouling, Flux decline, FO membrane cleaning, Spirulina, Draw solution, Biofouling

## Abstract

The forward osmosis (FO) desalination process has recently acknowledged a lot of attention as a promising solution for reducing the disadvantages of existing desalination systems. This work aimed to investigate the effect of a selected liquid organic fertilizer a novel draw solution produced from “microalgae *Spirulina platensis*” on the biofouling mechanism of FO membrane. Different draw solution (DS) concentrations ranging 240–480 g/L were examined, obtained water flux ranging from 6.5 to 3.4 Lm^2^h^-1^. A high flux decline was observed when using higher DS concentrations due to fouling layer accumulated throughout the membrane area which lowers the effective osmotic pressure difference. Different cleaning strategies were examined. The biofouled membrane was cleaned on-line with deionized water (DI) and externally using ultrasound (US) and HCl. Baseline experiments were done to investigate the efficiency of the cleaning strategies. After cleaning using the deionized water (DI) water, it was found that the water flux progressed from 3.4 to 7 Lm^2^h^-1^, while when using acid cleaning the flux recovered to 15 Lm^-2^h^-1^. The efficacy and amount of foulant removed by each cleaning stage were assessed using scanning electron microscopy (SEM) and energy dispersive X-ray spectroscopy (EDX).

## Introduction

Due to the global water crisis, many arid countries are utilizing desalination process to overcome the local water shortage. Recently, researchers focused on forward osmosis (FO) process as an efficient desalination technique with low energy consumption. One of the FO applications that provides a feasible substitute water supply for irrigation is fertilizer-drawn forward osmosis (FDFO). Adopting this technology within the Water-Energy-Food nexus holds great promise in providing desalinated water for food production while using less energy and having less environmental effect. FDFO is resilient and useful for a broad range of procedures in remote areas since it is a low-energy technology that is able to be operated by renewable energy (Al Bazedi et al. 2022; Amin et al. 2021; Nasr and Sewilam 2015a, 2015b).

In general, the FO process is established on the osmotic pressure of an extremely concentrated draw solution, which presents a driving force along the membrane, directing the flow from the feed solution to the draw solution. The FO process has a reduced fouling proclivity and a higher fouling reversibility than that of reverse osmosis membranes.

The operating conditions, which are comprised of hydrodynamic characteristics such as water flowrate, temperature, and recovery and cross-flow velocity, have a considerable influence on the effectiveness of the FO separation process. The boundary layer thickness along with the concentration polarization (CP) can be reduced through increased flow rates of the solutions between the membranes. Concentration polarization (CP) phenomena can be external (ECP) and internal (ICP). ECP can indeed be mitigated by increasing cross-flow velocity to establish a turbulent flow regime. However, ICP reduction is very challenging and depends on many parameters mostly the structural parameter of the membrane *S* (m) and the diffusivity of the draw solute. This, in its turn, reduces membrane fouling and improves water recovery (Ge et al. 2012a, 2012b). Coday et al. (2015) compared asymmetric cellulose triacetate against polyamide thin film composite for investigating the effects of membrane type and operating conditions on the FO functioning in producing desalinated water. This was done with system operating conditions similar to industrial operating parameters used for spiral wound reverse osmosis membranes. The efficiency of the FO procedure in the treatment of oil–water emulsions was explored by Duong and Chung (2014), where a variety of oil–water emulsion concentrations were examined up to 200,000 mg/L, and the FO process was proven to be very efficient in this study.

Different investigations were conducted on determining the FO draw solutions with the highest potential (Long et al. 2018; Johnson et al. 2018). The studies showed that the selection of suitable DS depends on several parameters including being affordable, non-toxic, and non-corrosive, efficient to create a high osmotic potential difference, have a low reverse solute flux, and also inexpensive. Substantial studies were carried out on comprehending the phenomenon of concentration polarization (Ryu et al. 2020), while others focus on creating innovative FO membrane production procedures and FO membrane fouling (Chun et al. 2017a, 2017b). Due to permeate flux decline as well as an observed decline in osmotic pressure, Zhao and Zou (2011) examined the effect of the rise in temperature on flux performance and recovery. The results showed that larger temperatures showed better initial flux values, increased water recovery, and higher concentration factors, but they also had negative impacts including elevated membrane scaling tendencies and more cleaning cycles. While Xie et al. (2013) measured the rate of clean water flux throughout the membrane for different FO membranes, the outcomes showed that water permeability increased with time for the tested membranes.

Other operating factors, e.g., feed water recovery and temperature, have an impact on the FO membrane’s performance. Temperature needed to be controlled, as it is acknowledged as a vital operating condition linked to different parameters including mass transfer, membrane fouling, concentration polarization, and salt solubility as studied by (Xie et al. 2013; Ge and Chung 2013).

Concentration polarization varies according to the FO system operation mode. There are two modes of operation in the FO system, when the active layer of the membrane is exposed to the feed solution, the membrane enters the FO operating mode and the membrane then shows external concentration polarization. While pressure retarded osmosis (PRO) operating mode is applied when the feed solution faces the membrane support layer, the membrane then shows dilutive external concentration polarization (Zhao and Zou 2011; Suh and Lee 2013).

As the solute is collected on the surface of the membrane within the feed solution side, it causes elevated concentration than in the bulk section. Permeation removes solute along the membrane’s surface (cell wall) in the draw side causing dilution of the solution (Phillip et al. 2010). Concentration polarization happens mutually on the sides of the forward osmosis membrane, where the “external concentration polarization (ECP)” and “internal concentration polarization (ICP)” occurred depending on the membrane orientation. Thereafter, the “concentrative” and “dilutive” external concentration polarization can occur in a forward osmosis process (Touati and Tadeo 2016).

Different variables affect the progress of a biofouling layer along the FO membrane surface comprising chemical and hydrodynamic variables, represented in permeation drag, calcium binding, and shear effect. Research and publications on FO membranes fouling employed organic foulants comprising humic acid, sodium alginate, and bovine serum albumin which are not considered a typical of true foulants found in natural water. Also, a typical activated sludge FS (feed solution) was utilized to conduct fouling investigations on the FO membrane (Chun et al. 2020; Mahto et al. 2021; Zhang et al. 2012).

Although the tendency for fouling in forward osmosis is common compared to reverse osmosis as presented by Lee et al. (2010), fouling issue needed to be addressed to optimize membrane performance and to increase membrane lifespan. It was found that membrane fouling not only lowers permeate flow but also affects membrane performance, represented in feed water quality and raising operational costs.

Cornelissen et al. (2008) found that either “reversible” or “irreversible” membrane fouling were nonexistent during the operation when the active layer of the FO membrane was facing the activated sludge solution. This might be due to running at low flux levels, potentially below the threshold flux for membrane fouling.

Achilli et al. (2009) investigated the FO-MBR fouling propensity and discovered that fouling is possibly reversible later to a starting period of irreversible fouling; moreover, it could be reversed by backwashing of the FO membranes. According to Chanhee et al. (2012), raising the crossflow velocity can greatly minimize the flux drop in the FO process. When the crossflow velocity was raised from 8.5 to around 34.2 cm/s, the results were promising, as the FO flux was improved.

There are two phases of membrane fouling comprising surface conditions and interactions between AL and foulants. Flux decline in the FO system is owing to the diminished osmotic pressure caused by cake formation along the membrane surface (Chun et al. 2017a, 2017b; Mi and Elimelech 2010).

The organic foulants are predicted to be deposited atop the membrane surface during the primary phase of the fouling mechanism, which elevates membrane hydrophobicity and reduces the filtration area causing hydraulic resistance of the membrane. Researchers explored and created several membrane cleaning approaches and processes in order to restore membrane flux. However, many of these cleaning methods may damage the membrane surface or it may be less practical, time consuming, and expensive. Furthermore, one of the key elements influencing membrane endurance is cleaning cycles. Ming et al. (2015) found that in the FO process gypsum scaling is largely recovered following the in-situ flushing, with a recovery rate of over 96%. Any changes in either the hydrodynamic parameters or the intermolecular adhesions have either no or slight influence on flux decline after the fouling layer develops.

Under diverse settings, the mechanisms and reversibility of blended organic–colloidal fouling employing silica and alginate colloids in FO system were comprehensively examined. When exposed to physical cleaning by increasing the cross flow, fouling layers produced during mixed organic/colloidal fouling runs under applied hydraulic pressure (PRO) were demonstrated to be irreversible. Those produced during normal FO operation, on the other hand, were found to be reversible to a large extent (Kim et al. 2014).

Organic fouling or “biofouling” in the forward osmosis process is difficult process affected by several factors, including membrane type, operating parameters, and type of feed and draw solutions. Various studies investigated the effectiveness of cleaning techniques for the elimination of organic fouling in the FO membrane, as well as recommendations of potential cleaning technologies for example ultraviolet and ultrasound methods. The most efficient way for restoring water flow in the FO process was discovered to be a sequence of physical and chemical cleaning (Yadav et al. 2020).

Physical cleaning alone proved to be ineffective for the FO process in recovering the decreased flow due to biofouling of both organic and inorganic fouling. Chemical cleaning with chlorine, on the other hand, proved quite successful for biofouling control (Yoon et al. 2013).

Unlike the RO process, FDFO is powered by the osmotic gradient between the feed and draw solutions, requiring little or no hydraulic pressure; FDFO has an excellent potential in many FO applications. The FO process’ performance is influenced by both (DS) and membrane properties. As a result, selecting the appropriate DS is crucial for the process efficiency (Phuntsho et al. 2013; Kim et al. 2017; Nematzadeh et al. 2020).

Inorganic fertilizers’ draw solution (DS) application for FO process has been studied by different researchers. Nasr and Sewilam (2015a, 2015b) investigated the utilization of FDFO desalination in brackish groundwater desalination in Egypt for environmental sustainability. The performance of (NH_4_)·_2_SO_4_ as a draw solution was investigated showing an effective process utilizing thin-film composite membranes (TFC), along with the high osmotic pressure of the draw solution, depleted reverse solute, and high feed solute rejection.

Most of the research regarding FDFO includes inorganic fertilizers (Phuntsho et al. 2012, and Phuntsho et al. 2013; Bagheri et al. 2019; Nasr and Sewilam 2015a, 2015b), while organic fertilizers are rarely addressed by researchers. Nematzadeh et al. (2020) studied an organic metal salt combination. They investigated the use of lended amino acids–metal–salts fertilizer DS and its performance via forward osmosis. The efficiency of employing an arginine-ZnCl_2_ fertilizer as a draw solution in Caspian Sea water desalination was also demonstrated. A variety of organic–inorganic complexes such as EDTAMgNa_2_ with good solubility, average molecular size, extended molecular structure, and suitable osmotic pressure were studied. These organic–inorganic compounds were discovered to have favorable water flow as well as a simple recovery procedure. Table 1 presents different draw solutions used in the FDFO process.

**Table 1:** Different draw solutions for FDFO process

Draw solute	Concentration (molar)	Osmotic pressure	*J*_w_ (LMH)	Reference
NH_4_HCO_3_	0.67	28 (atm)	7.3	Ge et al. (2013)
NH_4_CL	0.8	41.45 (atm)	3.61	Achilli et al. (2010)
KCl	2	89.3 (atm)	22.6	Ge et al. (2013), Phuntsho et al. (2012)
NH_4_NO_3_	1	33.7 (atm)	2.13	Phuntsho et al. (2012)
ZnCl_2_ + Arg	1:1	17.49 ± 2.86 (atm)	29.57	Nematzadeh et al. (2020)
EDTA-MgNa_2_	1	3 (atm)	9	Zhao et al. (2016)
Sucrose	1	26.7 (atm)	12.9	Ge et al. (2013)
*Spirulina platensis*	1.23	12.8 (atm)	7.5	Al Bazedi et al. (2022)

Organic fertilizers display less acidic properties in their aqueous solutions, which allows this type of fertilizers to be more proper than the inorganic fertilizers. Studies showed that organic fertilizers have higher salt rejection than the inorganic fertilizers. Also, the rate of reaction is slow in organic compounds, so it will not easily react with the feed solution (FS) (Corzo et al. 2017). Organic fertilizers can enrich the soil and prevent from diseases and deficiencies. The nutrients provided are not leachable and not dissolve either. More effective and better for the environment. The organic fertilizer release gradually nutrients to the plant which allow a contribution to the soil for several months.

The microalgae *Spirulina platensis* was studied as a possible fertilizer by different researchers (Masoud Alaa et al. 2012; Aung 2011; Faheed and Fattah 2008; Dineshkumar et al. 2018; Sridhar and Rengasamy 2002). From an agriculture point of view, microalgae *Spirulina platensis* can improve soil structure by increasing the amount of organic matter and stimulate the activity of soil microorganisms (Masoud Alaa et al. 2012; Aung 2011; Faheed and Fattah 2008; Dineshkumar et al. 2018; Sridhar and Rengasamy 2002; Jimenez et al. 2020). Wuang et al. (2016) explored the usage of spirulina as a fertilizer in combination with aquaculture effluent. The findings demonstrated a considerable increase in the growth rate of green vegetables as well as the dry weight of the seedlings.

According to the experimental work by Al Bazedi et al. 2022, *Spirulina platensis* shows a potential as draw solution for FDFO process. The primary goal of this study is to investigate the biofouling and the cleaning mechanisms while employing *Spirulina platensis* as a draw solution for FO.

## Materials and methods

### Membrane setup

**Fig. 1:**
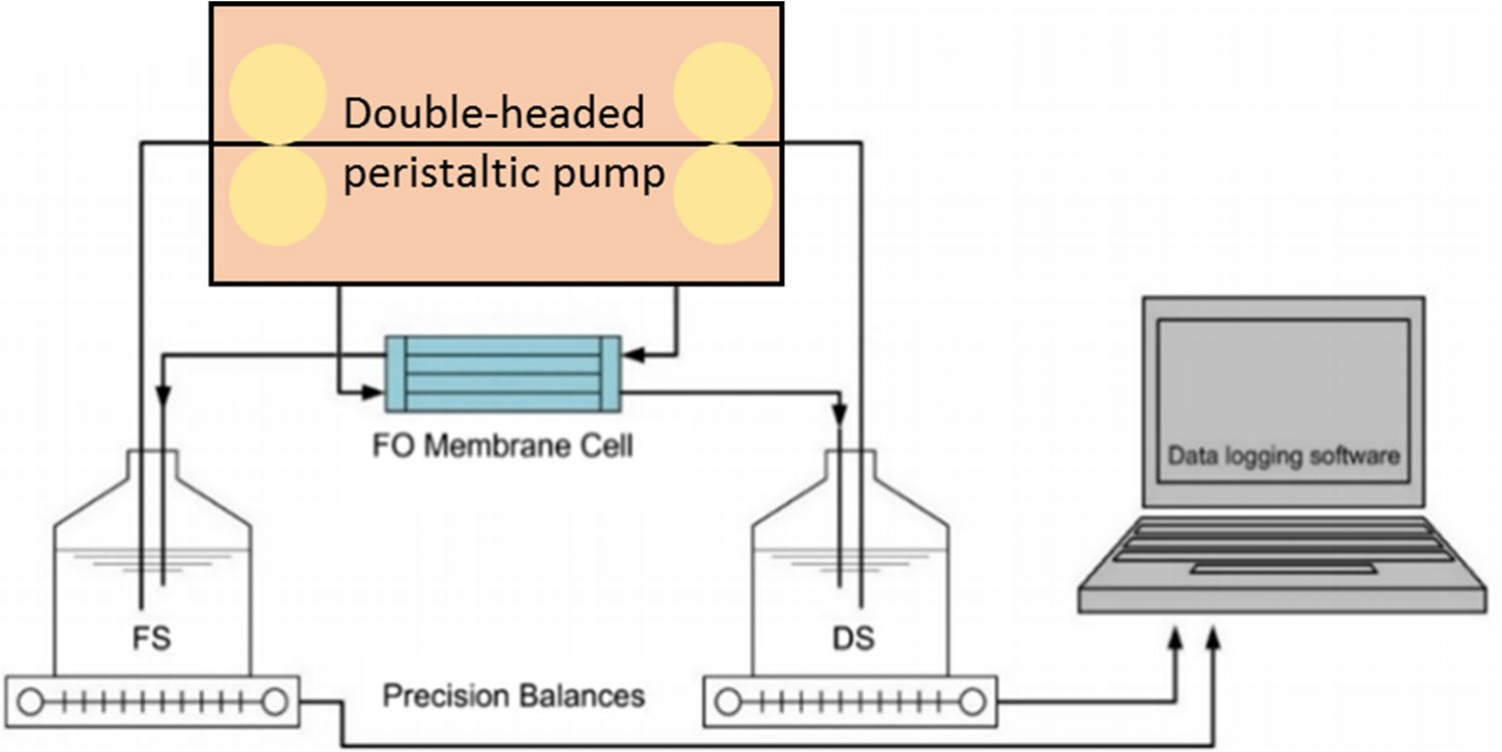
Experimental set-up (fluxometer)

The work in this paper were carried out by means of a lab-scale unit as displayed in Fig. 1. The system comprises a FO membrane with a total area of approximately 1.257 × 10^-3^ m^2^, and of a flow rate of 200 mL/min. The membrane sheet and the fluxometer were both procured from Porifera Inc., USA. Throughout the experiments, the FO mode of operation was adopted, with the draw solution facing the support layer and the FS facing the membrane active layer. The membrane characteristics are presented in Table 2.

**Table 2:** Forward osmosis membrane characteristics, Porifera Inc. (2015)

Item	Specification
Water permeation	FO mode: 33 ± 2 LMH PRO mode: 58 ± 3 LMH
Reverse salt flux	FO/PRO mode: 0.25 ± 0.1 g/L
Membrane structural parameter (*S* value)	215 ± 30 micron
Max trans-membrane pressure (TMP)	180 psi
pH operating range	2–11
Max chlorine	< 0.1 ppm
Max operating temperature	70 °C
Pure water permeability coefficient A (Lm^-2^ h^-1^ bar^-1^)	2.2 ± 0.01
Salt permeability coefficient of active layer, *B* (m/s)	1.6 × 10.7

### Organic fertilizer draw solution (OFDS)

Filamentous cyanobacterium microalgae *Spirulina platensis*, which is capable of growing in a variety of culture medium and forming enormous populations levels in tropical and subtropical ecosystems, e.g., lakes and reservoirs with high amounts of carbonate, bicarbonate, with pH values of up to 11, is used in the current study. Then, 1 kg of fresh algae was cut into tiny pieces and passed through a dual muslin cloth to remove waste. The various concentrations used in this study were created DI water addition.

The organic fertilizer draw solution (OFDS), which mostly consisted of spirulina, was made in concentrations varying from 240 to 480 g/L. Table 3 shows the main features of the organic DS. The Algal Biotechnology Unit, NRC, Egypt, is the source of fresh algae.

**Table 3:** Organic fertilizer characteristics

Component	Wt.%
Spirulina	96.35
CaO	0.4
MgO	0.25
K_2_O	1.5
P_2_O_5_	1.5

### Chemicals

Sodium chloride (analytical grade NaCl, 99%) obtained from ADWIC company-Egypt was used in these studies. To confirm that all of the salt was properly dissolved, proper mixing of the feed using a magnetic stirrer at 300 rpm. The experiments were performed at temperature of 25 °C. Table 4 shows the specifications for analytical grade NaCl.

**Table 4:** Standard specifications of analytical grade sodium chloride

Specification	Value
Molar mass	58.44 g/mol
Melting point	801 °C
Density	2.16 g/cm^3^
Solubility in water	Solubility in water: 360 g/L (20 °C)
Purity (%)	99%
Bromide	≤ 0.01%
Bromine (Br)	100 ppm max.
Calcium (Ca)	≤ 0.002%
Chlorate and nitrate	≤ 0.003%

**Fig. 2:**
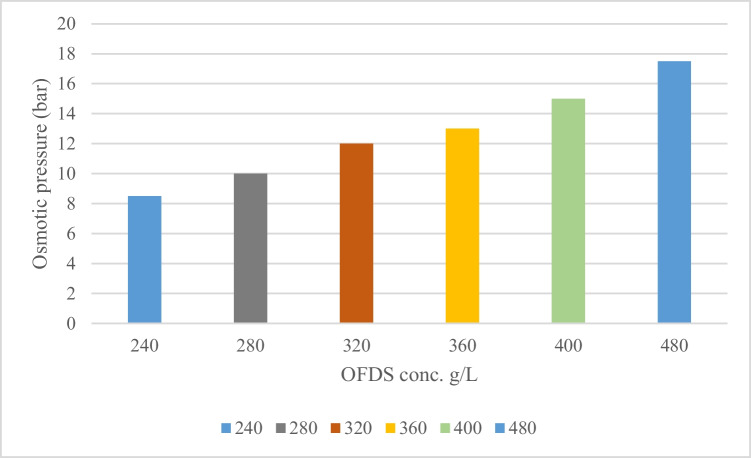
Osmotic pressure of different DS concentrations

The feed solution (FS) adopted concentration for these experiments was 10 g/L of NaCl. This concentration represents high brackish groundwater salinity level. Five concentrations of draw solutions (DS) were investigated ranges from 240 to 480 g/L of the selected organic fertilizer. The OFDS’s osmotic pressure was determined with an osmometer, as illustrated in Fig. 2. The FS was formulated by dissolving NaCl in DI water uniformly mixed by means of a magnetic stirrer at 300 rpm to verify that all salt was properly dissolved. The experiments were conducted at ambient temperature in an air-conditioned room at 25 °C.

### Methods

Water fluxes across the FO were calculated according to the change in feed solution volume. An online digital mass scale connected to the membrane setup (fluxometer), collecting and recording all the data concerning any difference in water volume (repetitively) at 2-minute periods. The water flux “*J*_w_” calculated using the equation (in Lm^-2^h^-1^) below.

1 $${J}_{\textrm{w}}=\frac{\Delta V}{(A)\ast (T)}$$

where ∆*V* is the total volume from FS to the DS (L), *A* is the area of membrane (m^2^), and *T* is the time (hr).

Reverse salt flux is caused by the ion transfer from the DS side to the FS side, which lowers the osmotic pressure difference between the membrane sides and therefore reducing the driving force. The reverse solute for each fertilizer DS varies greatly dependent on the solute properties. The reverse permeability of the DS consists of ions with larger hydrated diameters was lower than that of DS-containing ions with smaller hydrated diameters. Solute from the DS to the FS reverse diffusion is predicted owing to the concentration difference between them (Al Bazedi et al. 2022; Amin et al. 2021; Nasr and Sewilam 2015a, 2015b). Final DS analysis were conducted at the Agricultural Research Center (ARC) in Cairo.

2 $$\textrm{RSF}={J}_{\textrm{s}}=\left(\textrm{Vi}-\varDelta V\right)\ast \textrm{Cs}/\textrm{Area}\times \textrm{Time}$$

where Vi is the initial FS volume, Cs is the final concentration of the draw solute in the FS at the end of the experiment, and Δ*V* is the total volume of water that enters the DS from the FS.

The osmolality, osmotic concentration (Osmol/Kg) of various concentrations of DS used in the experiments, was determined using an osmometer (Osmomat 030, cryoscopic osmometer, Gonotec, at Cairo University). Using Eq. (3), the measured osmolality was then converted to osmotic pressure (atm) at a temperature of (22 °C ± 1 °C) [1].

3 $$\textrm{OP}=\textrm{RTc}$$

where OP is the the osmotic pressure (atm), RT (kg · atm/mol): 24.22 at 22°C, and *C* (moles/Kg) is the draw solution osmolality

The DS and FS both started out with a mass of 250 g. The water flux was calculated based on the flux/time graph, which showed a consistent flow for the first 15 mins of operation. The experiments continued at least two to three hours to guarantee sufficient DS diffusion and appropriate observing any reverse DS diffusion. Longer operation durations (testing time) resulted in lower flux (dilution of the draw solution) owing to the deposition of organic materials atop the membrane surface.

### Membrane fouling

The problem of membrane biofouling, which is usually an outcome of the adsorption of biofoulants along the membrane surface. It is primarily influenced by membrane surface properties and affects the initial fouling rate. However, in terms of membrane surface characteristic, the foulant–foulant interaction is difficult to identify since the clean surface has been already enfolded by foulants as a result of the original membrane–foulant contact. Membrane biofouling is examined by the measurement in flux decline as well as surface morphology using scanning electron microscopy (SEM) and energy dispersive X-ray spectroscopy (EDX), which is used to figure out the biofouling layers taking place on the membrane surface.

### Cleaning strategies

Throughout the FO operation, biofouling was studied. Different cleaning procedures were studied including cleaning with deionized water (DI) (online cleaning); cleaning with either ultrasound (ultrasonic cleaner set WUC-D 03H) or HCl (3%) were also proposed as part of the integrated cleaning regimens. The efficiency of the cleaning methods was evaluated. In addition, following each cleaning process, the membrane performance as well as membrane surface was examined.

## Results and discussion

### Biofouling mechanism and flux decline

**Fig. 3:**
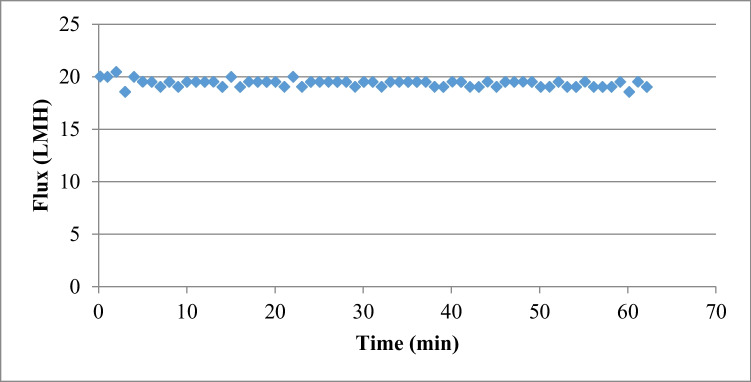
Base experiment using 1 molar NaCl

**Fig. 4:**
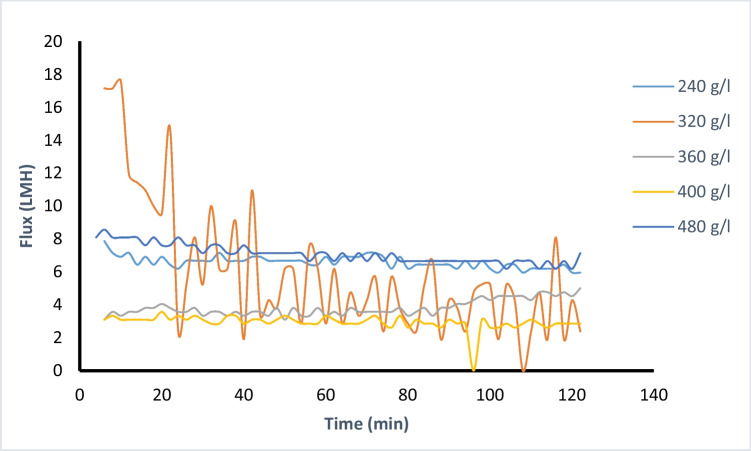
Flux decline of different DS concentrations

In the baseline experiments, DI water was employed as the feed solution for the process. A Total Dissolved Solids (TDS/EC) meter (Orion (Thermo Scientific) is used to measure total dissolved solids (TDS) before and after each experiment. Baseline experiments were employed before and after each experiment to study the fouling impact on the water flux as displayed in Fig. 3. *Spirulina platensis* solution was chosen as the draw solution for this study. The main aim of this selection is that the *Spirulina platensis* algae contained in the DS have potentially an elevated osmotic pressure, plus the fact, that the molecule of an organic matter is big so we can avoid reverse solute diffusion. Figure 4 shows the flux of different OFDS concentration.

The major cause of the rapid drop in water flow is the concentration polarization (CP) phenomenon; as a drop in the net driving force is occurred through the membrane surface (Lee et al. 2010). The observed limitation in the water flux presented in Fig. 4, is attributed to a reduction in the effective osmotic pressure affected by the build-up of concentration gradients across the membrane.

Different draw solution concentrations ranging 240–480 g/L were examined; obtained average water flux ranges from 6.5 to 3.4 Lm^-2^h^-1^. The observed biofouling mechanism may be categorized in two stages of fouling. The primary stage in which foulant is adsorbed on the clean membrane surface (initial rapid fouling). In this initial step, the biofouling rate is critical to overall efficiency. The buildup of foulant–foulant atop the membrane surface consequently represents the following stage in the biofouling mechanism which can be represented as a long-term cumulated biofouling. It is presented in Fig. 4 that for DS solution with concentration 240 g/L, the starting flux was 6.4 Lm^-2^h^-1^ after 15 min of operation, while the lower flux reached was 5.9 Lm^-2^h^-1^ after 2.5 hrs. There was a sharp flux decline at DS concentration of 280 and 320 g/L compared to higher DS concentrations. This rapid decline was assumed to be due to the initial rapid fouling mechanism.

For higher DS concentrations starting from 360 g/L, the maximum flux observed was 3.3 Lm^-2^h^-1^ after 15 min of operation; this is owing to foulants accumulating on the membrane surface. It was comprehensible that the primary biofouling stage is much rapid because to the high concentration of organic fertilizer used. The second stage of biofouling, which comprises the foulant buildup on the membrane surface is of the same pattern for each of the DS concentrations (Wuang et al. 2016). The non-linear flux performance with the rising in draw solution concentrations is ascribed to this concentration polarization CP effect. The formation of concentration gradients reduces both *J*_w_ and *J*_s_ by decreasing the effective osmotic pressure difference and the difference in solute concentration across the active layer. Also, pore clogging of the porous membrane lower the effective pressure (Charlton et al. 2021). The CP effect is attributed to external concentration polarization at the DS side (Bhinder et al. 2017). The CP is the main reason for the notably water flux reduced rates in comparison to other studies using inorganic fertilizers (Nasr and Sewilam's (2015a, 2015b); El Zayat et al. 2021).

A minor pressure variance between both the FS and DS solutions accounts for minimal flow measured. The observed flow is significantly lower than Nasr and Sewilam's (2015a, 2015b) inorganic DS utilizing the same membrane type. While, when compared to other organic DS, the findings are in accordance with the findings of Wang et al. (2017), when using a tailored-made TFC membrane, and also the conclusions of Alaswad et al. (2018), where they studied the performance of DS solution of sucrose and glucose versus DI as FS using a NF membrane (TFC-SR2) in FO mode as the FS faces the active layer in a forward osmosis (FO) system (Wang et al. 2017; Alaswad et al. 2018)

Gwak et al. (2015) revealed that low molecular weight DS solutions higher water flux is affected by its reduced viscosity and elevated solubility. Furthermore, Long and Wang (2015) studied carboxy ethyl amine sodium salts as DS because of their large molecule sizes and comparatively high osmotic pressure; the results showed that in PRO mode both increased water flux and decreased reverse solute flux were achieved.

**Fig. 5:**
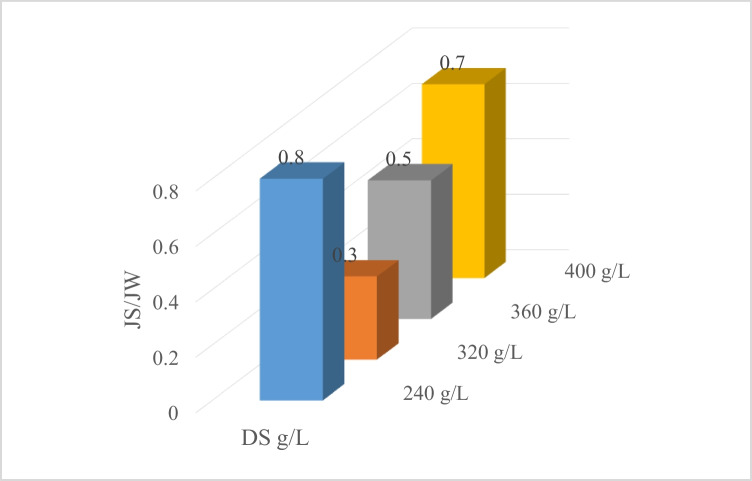
FS/DS concentration membrane selectivity *J*_S_/*J*_W_

No reverse flow from DS was identified due to the biofouling and the fouling layer formation. Figure 5 shows the membrane selectivity *J*_S_/*J*_W_ determined for various DS concentrations. The computed *J*_S_/*J*_W_ are between 0.1 and 0.8 intended for various DS concentrations, owing to high DS concentrations which result in high ICP impact. The results are in accordance with the results of Bowden et al. (2012), an elevated specific reverse solute flux (SRSF) evaluated to be 0.65 g/L in FO for organic Goh et al. (2022) measured SRSF 0.1877 g/L for PEG-400 in acetone. Because reverse solute flux (RSF) represents an inherent property of the membrane’s selective layer, it is altered in response to different organic solvents properties.

The change in feed and draw solution concentration were calculated by analyzing the final concentration of each showing Na^+^ in feed solution is concentrated by 41%, Cl^-^ by 36%, and spirulina is diluted by 20%.

### Cleaning approach

The effect of physical cleaning on the OFDS’s performance suggests that the biofoulants formed at the surface of the membrane are reversible. Any absence of “hydraulic pressure” in the forward osmosis process leads to a dense and broad layer of organic biofouling on the membrane surface, which is eliminated by creating shear stress, as evidenced by the cleaning techniques' efficacy (Wang et al. 2015).

Different cleaning techniques were investigated. Online cleaning using DI water 1 hr. Also, cleaning using HCl solution (3%) at pH 6 for 2 min, then online cleaning using DI water for 20 min was investigated. The cleaning efficiency was measure through the recovery of flux.

Osmotic backwashes were evaluated on fouled membranes after each fouling experiment. During osmotic backwash, the feed water was replaced by a solution of NaCl with 29 g/L concentration, and the draw water was replaced with DI water (to allow for water penetration in the opposite direction of the typical FO operation) for 15 minutes.

**Fig. 6:**
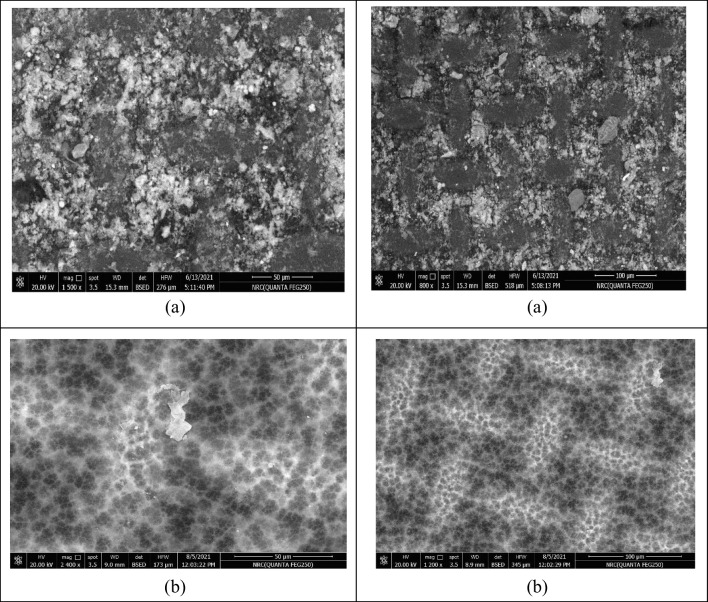
Membrane SEM images: **a** fouled membrane and **b** membrane after acid cleaning

Biofouling was more severe when using the Porifera FO membrane with organic fertilizers as DS; therefore, osmotic backwash was also investigated for 1 hr. Though when osmotic backwashing was used, the foulant cake was only partially eliminated. A scanning electronic microscope (SEM) was used to assess and evaluate the fouling in the membrane, as well as the cleaned membrane showed in Fig. 6.

**Fig. 7:**
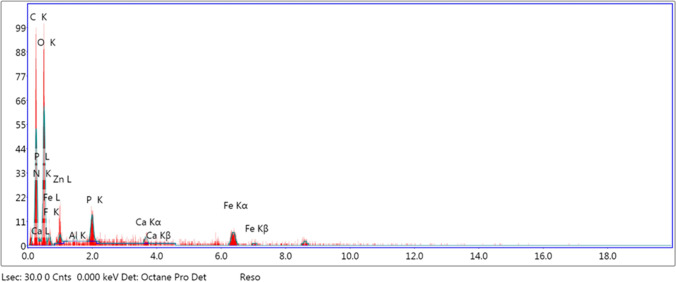
EDX of fouled membrane

**Fig. 8:**
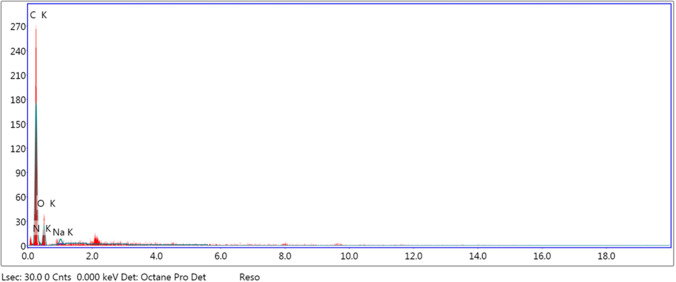
EDX of acid cleaned membrane

The SEM images showed the presence of some organic crystals deposited to the membrane surface as shown in Fig. 6a. The constitution of foulants was examined by EDX (Figs. 7 and 8). Figure 7 represents the biofouled membrane, while Fig. 8 represents the acid cleaned one. By comparing the results in both Figs. 7 and 8, it is clear that most of elements deposited on the membrane presented in Fig. 7 are eliminated or reduced by using acid cleaning, which is clearly presented in Fig. 8.

**Fig. 9:**
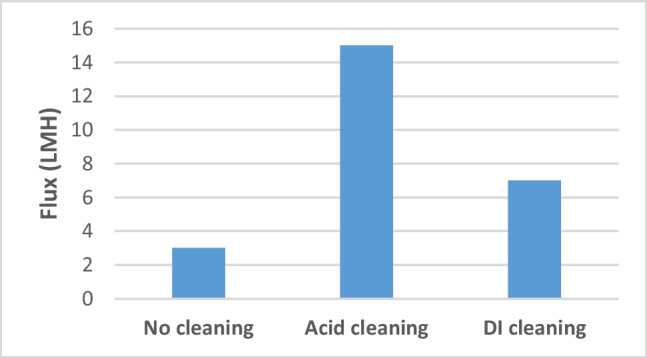
Efficiency of different cleaning methodologies on water flux recovery

The efficiency of using DI water in cleaning protocol on the water flux recovery was presented in Fig. 9. The membrane performance was investigated using baseline experiment for both cleaning methods. The water flux was reduced to reach 2.5 Lm^-2^h^-1^. After cleaning using DI water, it was seen that the water flux is improved from 3.4 to 7 Lm^-2^h^-1^, while when using acid cleaning the flux is recovered to 15 Lm^-2^h^-1^. Therefore, the cleaning using DI water only remained to be not adequate to achieve a reasonable flux improvement. Ultrasound using ultrasonic cleaner set WUC-D 03H was examined as a cleaning mechanism at 40 kHz. The impact of ultrasonic irradiation on flux recovery was studied. The membrane displays negligible flux recovery after 5–10 mins. When the membranes were irradiated directly out of the module after 20 mins, they were damaged and huge holes were developed due to lingering in the direct sonic cavitation area.

**Fig. 10:**
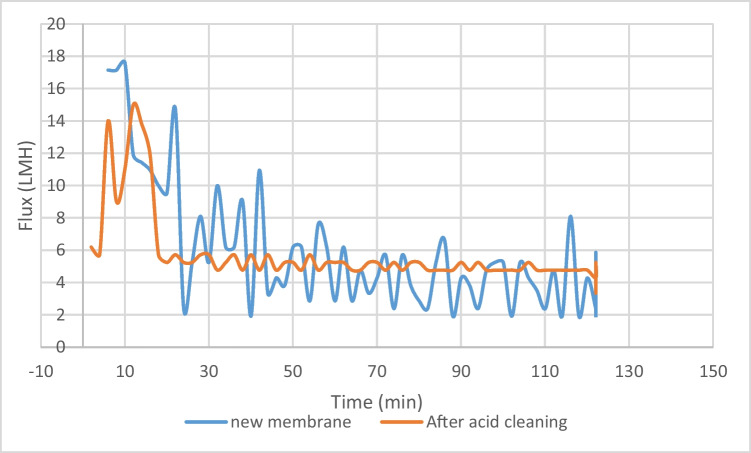
Flux decline of DS 320 g/L, before and after acid cleaning

**Fig. 11:**
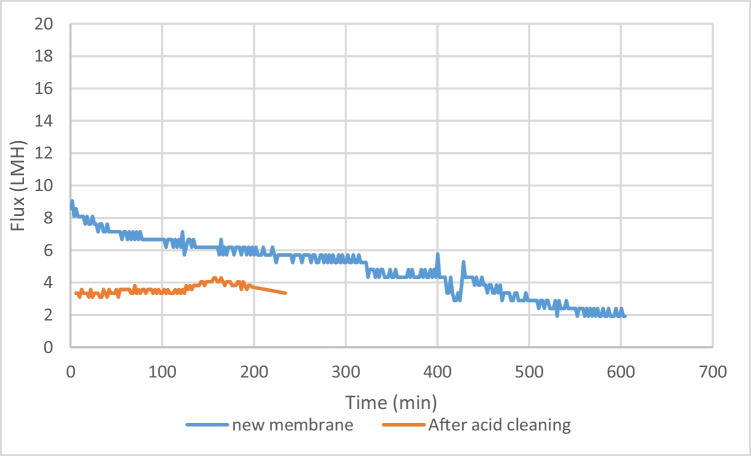
Flux decline of DS 480 g/L, before and after acid cleaning

Two DS concentrations were chosen to study the effect of acid cleaning on the membrane performance. Figures 10 and 11 show the flux decline along the process. Figure 10 shows the flux decline of DS concentration 320 g/L, as this concentration showed the higher flux along all the other concentrations. The average flux for clean membrane is 5.86 Lm^-2^h^-1^, while for acid cleaned membrane is 4.59 Lm^-2^h^-1^.

Figure 11 shows the flux decline of DS concentration 480 g/L, as this concentration was the highest concentration used. The membrane’s performance displays various flux drop stages while operating, the first after 62 mins and the latter after 225 mins of operation, as illustrated in Fig. 11. The final flux-drop phase was seen between 510 and 610 mins of operation, resulting in a minimum flux value of 1.9 L/min (m^2·^h). The reduced pressure difference across the membrane surface caused a decrease in flux and water flow. Additionally, the ICP presented on the membrane surface reduced the water flow (Zou et al. 2011). After acid cleaning, 50% of the membrane flux was recovered, showing an average flux of 3 Lm^-2^h^-1^. For acid cleaning protocols, it was seen that the membrane deteriorated after 3 cleaning cycles. No reverse flux was observed, which attributed to the biofouling mechanism as well as pore clogging due to large DS molecules.

## Conclusion

Spirulina microalgae is a relatively inexpensive organic fertilizer which may be used in a FO system as a DS. In this study, *Spirulina platensis* flux performance and the biofouling mechanism of the FO membrane for different Spirulina concentrations was investigated.

“Organic fouling” or biofouling is a complicated process in forward osmosis, and it is impacted by a various factors, including but not limited to the type of feed and draw solution, operating parameters, and membrane type. Membrane biofouling is influenced by means of the combined impact of chemical and hydrodynamic interactions. The influence of different organic draw solutions on biofouling behavior with different FS is not well documented in different resources, and additional studies are needed in this area. Different draw solution concentrations ranging 240–480 g/L were examined; obtained water flux ranges from 6.5 to 3.4 Lm^-2^h^-1^. According to the findings, the primary mechanisms influencing the biofouling layer formation atop the membrane surface; including permeation drag, the organic matter binding, and the hydrodynamic shear force along the membrane area as well as membrane pore clogging. The results implies that foulant–foulant interaction is critical in influencing the frequency and degree of organic fouling.

The organic fertilizer used (microalgae *Spirulina platensis*) has a strong intermolecular interactions and shear force due to calcium binding (according to the EDX results), in addition a cake layer is formed under the tested conditions as presented in the SEM. The flux behavior acts non-linear among any rising in the DS concentrations, which is credited to the concentration polarization CP occurrence. The CP is the main cause of the drastically low water flux rates contrasted to other studies using inorganic fertilizers. Moreover, the results validate the possibility of using organic fertilizers as a DS for direct FO desalination applications. Additional research towards preventing membrane biofouling caused by organic fertilizer as well as studying the financial feasibility of the proposed system.

## Data Availability

The datasets used and/or analyzed during the current study are available from the corresponding author on reasonable request.
